# Influence of Tilting Angle on Temperature Measurements of Different Object Sizes Using Fiber-Optic Pyrometers

**DOI:** 10.3390/s23198119

**Published:** 2023-09-27

**Authors:** Salvador Vargas, Alberto Tapetado, Carmen Vázquez

**Affiliations:** 1 Electrical Engineering Faculty, Universidad Tecnológica de Panamá, Ave. Universidad Tecnológica, El Dorado, Panamá 0819-07289, Panama; salvador.vargas@utp.ac.pa; 2 Electronics Technology Department, Universidad Carlos III de Madrid, 28911 Leganés, Spain; atapetad@ing.uc3m.es

**Keywords:** modeling, optical fiber sensor, pyrometer, temperature, tilting angle, finite hot spot

## Abstract

This article presents a new model of optical power gathered by a fiber-optic pyrometer when there is a tilting angle between the fiber longitudinal axis and the vector perpendicular to the tangent plane of the emitted surface. This optical power depends on the fiber specifications, such as the diameter and the numerical aperture (NA), as well as the object parameters, including its diameter, emissivity, and tilting angle. Some simulations are carried out using other pyrometers from the literature without tilting to validate the model. Additional simulations with different optical fibers, object sizes, and distances at different tilting angles allow us to describe the behavior of the pyrometer when the object is smaller than the optical fiber field of view (the light cone defined by its NA). The results show that for a finite surface object, the power collected by the optical fiber is affected by changes in the tilting angle, greater tilting lesser gathered power, and reaching the maximum power when the field of view of the fiber covers up the entire object, as expected. On the other hand, additional equations are presented to describe the maximum tilting angle, and distance that allow the maximum power gathered for a determined object diameter and fiber, avoiding temperature measurement errors.

## 1. Introduction

Temperature measurement is essential in order to understand natural phenomena and many industrial processes [1]. To measure temperature over a large range above 1000 °C, techniques that need contact with the object, such as thermocouples, can be used. However, their installation is difficult or nearly impossible if any of the parts are in motion, and they have slow response times [2]. Another choice is to employ non-contact methods, such as Raman thermometry, infrared (IR) thermography with cameras, or pyrometry. IR cameras require the use of lenses and the existence of a viewing angle that allows measurement [3]. In addition, uncertainty when it comes to knowing the value of the object emissivity negatively affects the precision of this technique [4]. In the case of Raman thermography, exposure times are usually high and require the excitation of the sample in addition to processing the received signal [5]. The spatial resolution of this method depends on the size of the laser spot exciting the sample [6]. Two-color fiber-optic pyrometry, in addition to allowing rapid acquisition [7], high spatial resolution [8], and precision [9], can be used in environments with difficult access and in extreme conditions, highlighting designs without discrete optics that limit their performance [10]. A fiber-optic pyrometer allows temperature measurements without considering the effect of emissivity if at least two spectral bands are used, as in two-color fiber-optic pyrometers. Using two close spectral bands in combination with filters can reduce insertion losses and measurement errors [11,12]. Fiber-optic pyrometry is used in multiple applications, such as machining processes [13], rock friction monitoring [14], combustion engines [15], cutting with electrical discharges [16], bioengineering [17], etc. [18]. One benefit of this technology is its high spatial resolution, which relies on the size of the optical fiber employed and the distance between it and the target being measured [8]. For the first time, a standard single mode fiber (SMF) was used in [10], with a theoretical maximum spatial resolution of 16 µm for a target surface at 25 µm. A model to quantify the recovered radiation’s dependency on the object’s position and size is shown in [8]. Another author included the effect of a tilting angle between the normal to the emitting target and the axis of the fiber for an optical fiber field of view smaller than the object but not in the opposite case [19]. The tilting angle can be presented in applications where the object size is finite and smaller than the field of view. For example, in [20], a fiber-optic two-color pyrometer measured in situ nanoparticle cloud formation during the combustion of single micron-sized iron particles. However, the angle between micro-particle and fiber-optic was not considered. In another study [21], a two-color pyrometer measured the temperature of small, falling samples in a microgravity materials processing experiment. This work examines the advantage of tilting the collecting fiber to increase the time that the falling particle remains in the fiber field-of-view. Fiber-optic pyrometry monitors the conditions of burst of nuclear fuel claddings to prevent potential nuclear accidents. Different uncertainties on temperature measurements in small areas are analyzed in [22] but without the tilting effect. Some tests of fiber-optic pyrometers with high-spatial resolution rely on measurements with an optical fiber field of view smaller than the object [23].

In this work, a new model is presented that considers an angle between the axis of the fiber and the normal to the emitting target, regardless of the relationship between the size of the object and the field of view of the fiber, including when the object is smaller than the field of view for the first time. All the previous models present only the study of emitting targets of different sizes but without tilting angles, and the only one found with a tilting angle describes objects greater than the field of view of the fiber. Our model offers a practical advantage in situations where the alignment of the fiber is not precisely controlled or when, due to the usage, alignment drift exists, allowing the determination of the collected power and temperature of the object within a precision range, or inversely the tilting angle responsible for the mismatch in temperature. A software program that implements the model simulates and validates the pyrometer’s behavior and design parameters for different cases. Equations to find the permitted maximum tilting and distances and to avoid measurement errors are derived.

## 2. Theoretical Background and Modeling

This section presents the mathematical-geometric model of a fiber-optic pyrometer, which unlike those previously reported [8,19], includes an angle between the normal to the emitting target and the axis of the fiber for any target size. Initially, we provide a summary of the previous model [8] as a reference for the calculation process. Subsequently, we present a new model with the tilting angle and the new integration limits.

### 2.1. Fiber-Optic Pyrometer Aligned with Target Surface

**Figure 1:**
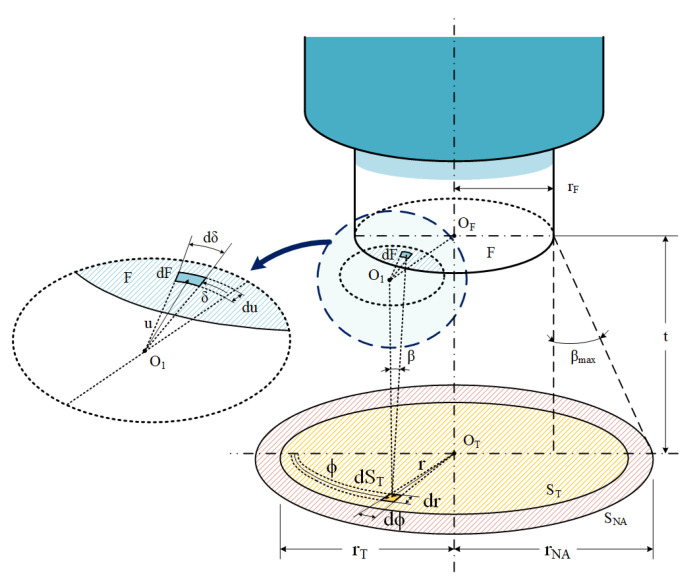
Schematic of a fiber-optic pyrometer aligned with the target surface, showing the model variables. Adapted from [24].

The model presented in [8] describes the light collected by a fiber-optic pyrometer from a circular target in which the center is aligned with the fiber axis, and the angle between the normal to the target and the fiber axis is zero degrees (see Figure 1).

This model includes two steps. First, it calculates the fiber coupled differential power (*P_dλ,dST_*) due to a differential element of the target surface (*dS_T_*). This is done by integrating the spectral radiance (Lambertian type) over the solid angle differentials generated by the surface resulting from the intersection of the two circles in the fiber end plane. One defined by the radius of the fiber core (*r_F_*), and another one defined by the cone projection of light from the differential element of the target to the end plane of the fiber, with radius *r_βmax_*. The cone half angle is the maximum acceptance angle of the optical fiber (*β_max_*), given by:

(1) $${\beta_{max}=sin}^{-1}\left(\frac{NA}{n_{0}}\right)$$

where *NA* is fiber-optic numerical aperture, and *n_0_* is external medium refractive index.

Then, the coupled spectral power is given by:

(2) $$P_{d\lambda ,dS_{T}}=\iint_{A_{F}\cap A_{\beta max}}^{}L\left(\lambda ,T\right)\cos\left(\beta \right)dA$$

where *L(λ,T)* is the spectral radiance of the emitting target, *λ* is the wavelength, *T* is the absolute temperature, *β* is the angle that the normal to *dS_T_* forms with each solid angle differential generated by the intersection of the circles, and *dA* is the solid angle differential (see Figure 1 and [8]).

After expressing the differential area (*dA*) in terms of the radial (*u*) and azimuthal (*δ*) cylindrical coordinates, the resulting expression is as follows [8]:

(3) $$P_{d\lambda ,dS_{T}}=\int_{u=u_{min}}^{u_{max}}\int_{\delta =\delta_{min}}^{\delta_{max}}L\left(\lambda ,T\right)\frac{{u\;t}^{2}}{{\left(t^{2}+u^{2}\right)}^{2}}d\delta du$$

where *t* is the distance from the object plane to the fiber end plane, and *u_min_*, *u_max_*, *δ_min_*, and *δ_max_* are the integration limits.

To define the integration limits, the distance between the centers of both circles and the relationship between *r_F_* and *r_βmax_* must be considered. This distance corresponds to the radial position (*r*) of the differential element (*dS_T_*), which is measured from the center of the emitting object surface. Based on the relationship between *r_F_* and *r_βmax_*, three integration cases can be distinguished. Once the appropriate case is selected, the radial position specifies the limits of *u_min_*, *u_max_*, *δ_min_*, and *δ_max_* regardless of its azimuthal position due to the symmetry of the resulting configuration with respect to this angle.

Finally, to find the total coupled spectral power, (3) is integrated over the entire surface of the emitting target (*S_T_*) [8].

**Figure 2:**
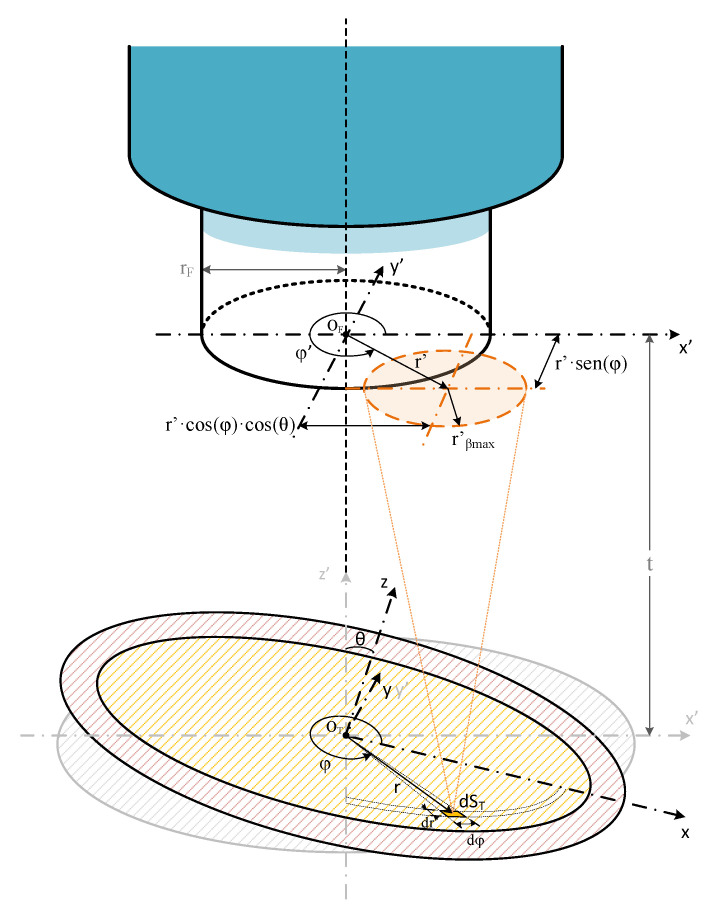
Schematic of the fiber-optic pyrometer with a tilting angle showing the model variables.

Table 1 lists the variables described in the text and used in Figure 1 with their meanings, along with those shown afterwards in Figure 2.

### 2.2. Fiber-Optic Pyrometer with a Tilting Angle to Target Surface

Now, the circular emitting target has its center aligned to the axis of the fiber, but the target has a non-zero tilting angle, denoted as theta (*θ*). It is the angle between the fiber axis and the normal to the emitting target surface (see Figure 2). This angle changes the equations of the previous model. However, the general procedure remains the same, i.e., calculating the fiber-coupled spectral powers (*P_dλ,dST_*) due to each target differential (*dS_T_*), and summing up all of those powers.

To account for the tilting angle, a new Cartesian coordinate system is introduced, with the *x* and *y* axes laying in the plane of the target surface, passing the *x* axis through the nearest and farthest points from the target to the fiber end plane, and the *z* axis normal to the target surface, as depicted in Figure 2.

Now, the shortest distance between the differential element *dS_T_* and the end plane of the fiber is not constant for all differentials *dS_T_*, unlike in [8]. The new distance *t’* depends on the radial and azimuthal coordinates of the differential and is given by:

(4) $$t^{\prime }=t+∆t=t+r\;cos\left(\varphi \right)sin\left(\theta \right)$$

where *t* is the distance from the fiber-end plane to the plane that passes through the center of the target and is parallel to the fiber-end plane, *r* and *φ* are the radial and azimuthal coordinates in the plane of the target, and *θ* is the target tilting angle.

**Figure 3:**
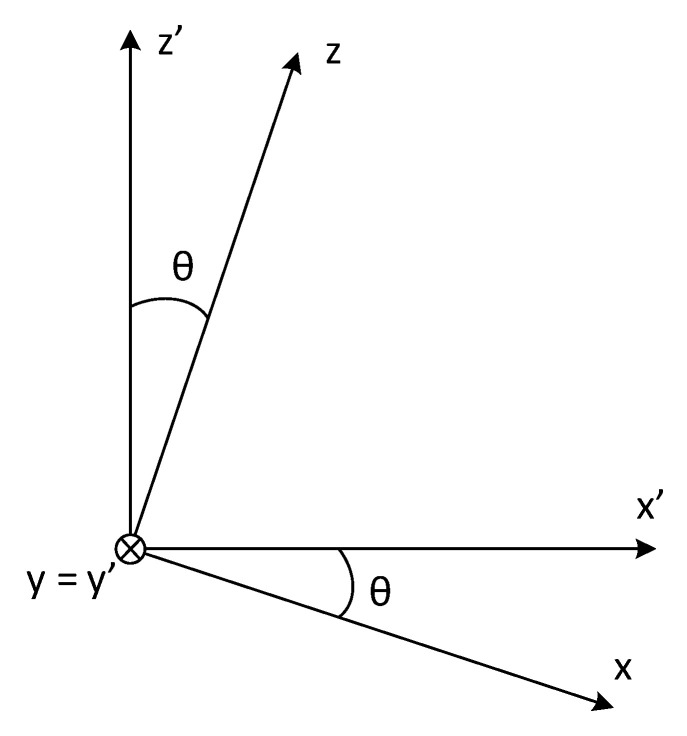
Cartesian coordinate systems (*x’*, *y’*, *z’*) and (*x*, *y*, *z*), and their relationship.

To find *P_dλ,dST_*, it is necessary to calculate *cos(β)*. This value is obtained from the scalar product between the unit normal vector of the target surface $\hat{V_{N}}$, with the unit vector defined from the differential *dS_T_* to the differential element of solid angle *dA*, named $\hat{V_{dS_{T}-dA\;}}$. These unit vectors are expressed over a new Cartesian coordinate system (*x’*, *y’*, *z’*), defined by rotating the *x-y* plane of the Cartesian coordinate system (*x*, *y*, *z*), an angle of *−θ*, using the *y* axis, which now coincides with *y’* (see Figure 3). The unit vector normal to the target surface is given by:

(5) $$\hat{V_{N}}=sin\left(\theta \right)\;\hat{x^{\prime }}+cos\left(\theta \right)\;\hat{z^{\prime }}$$

The unit vector from *dS_T_* to *dA*, is given by:

(6) $$\hat{V_{{dS}_{T}-dA\;}}=\frac{1}{\sqrt{u^{2}+{t^{\prime }}^{2}}}\left[u\;cos\left(\delta \right)\;\hat{x^{\prime }}+u\sin\left(\delta \right)\;\hat{y^{\prime }}+t^{\prime }\hat{z^{\prime }}\right]$$

where *u* and *δ* are the radial and azimuthal coordinates of the differential element of area that defines the solid angle differential *dA* over the cylindrical coordinate system.

The scalar product of (5) with (6) results in *cos(β)* given by:

(7) $$cos\left(\beta \right)=\frac{1}{\sqrt{u^{2}+{t^{\prime }}^{2}}}\left[u\;cos\left(\delta \right)\sin\left(\theta \right)\;+t^{\prime }\cos\left(\theta \right)\right]$$

After replacing (7) in (2) and expressing *dA* as a function of *dδ* and *du*, it is found that the spectral power coupled to the fiber by *dS_T_* is given by:

(8) $$P_{d\lambda ,dS_{T}}=\int_{u=u_{min}}^{u_{max}}\int_{\delta =\delta_{min}}^{\delta_{max}}L\left(\lambda ,T\right)\frac{\left[u\;cos\left(\delta \right)\sin\left(\theta \right)\;+t^{\prime }\cos\left(\theta \right)\right]\;t^{\prime }\;u}{{\left({t^{\prime }}^{2}+u^{2}\right)}^{2}}d\delta du$$

where *L(λ,T)* is the spectral radiance of the emitting target object, and *u_min_*, *u_max_*, *δ_min_*, and *δ_max_*, the integration limits.

### 2.3. Limits of Integration

To determine the integration limits, it is necessary to compare *r_F_*, the circle of light projected by the *dS_T_* on the fiber end plane, and the distance between the centers of both circles, as in the previous model.

Since each differential element *dS_T_* is now at a different distance *t’* depending on its position in the emitting target (see (4)), the projected circle by this element in the fiber end plane has a radius *r’_βmax_* given by:

(9) $${r\prime }_{\beta max}=t^{\prime }\times \tan\left(\beta_{max}\right)$$

where *β_max_* is the optical fiber maximum acceptance angle.

The distance between the centers of the circles with radii *r_F_* and *r’_βmax_*, defined in the end plane of the fiber, is different from the radial position of the differential element *dS_T_*. This new distance *r’* is given by:

(10) $$r^{\prime }=\sqrt{{\left(r\;cos\left(\varphi \right)cos\left(\theta \right)\right)}^{2}+{\left(r\;sin\left(\varphi \right)\right)}^{2}}$$

The distance *r’* and the relationship between *r_F_* and *r’_βmax_*, defines the intersection area of these circles and allows selection of the integration case. These are the integration of circles, arcs and circles, or just arcs, as in [8]. Now, each *dS_T_* differential has its respective integration case and integration limits.

The integration limits of *u* for (8) are shown in Table 2, where each cell specifies the limits that this variable has according to its integration case. When integrating circles and arcs, the integral is divided into the sum of two integrals, each one with their respective *u_min_* and *u_max_* integration limits.

The integration limits *δ_min_* and *δ_max_*, in (8), for the circumference integration case, are *0* and *2π* respectively, as in [8]. For arcs, the integration limits *δ_min_* and *δ_max_*, are given by:

(11) $$\delta_{min}=\varphi^{\prime }+\pi -\delta_{i}$$

(12) $$\delta_{max}=\varphi^{\prime }+\pi +\delta_{i}$$

**Figure 4:**
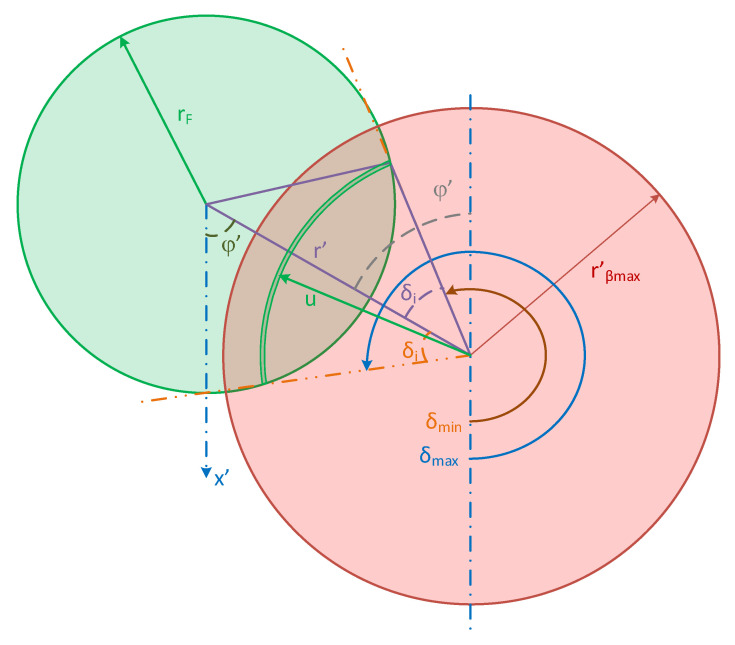
Geometry of the fiber end plane used to calculate the delta limits *δ_min_* and *δ_max_*, in the arc integration situation.

Figure 4 shows the geometry used to calculate these limits. They depend on two angles: the first is the half difference of the limits of integration *δ_i_,* and the second is the angle (*φ’*) between the vector *r’* and the *x’*-axis. *δ_i_* was already defined in [8] as:

(13) $${\delta_{i}=\frac{1}{2}\;\left(\delta_{\max}-\delta_{\min}\right)=\cos}^{-1}\left(\frac{{r^{\prime }}^{2}+u^{2}-r_{F}^{2}}{2r^{\prime }u}\right)$$

The angle *φ’*, is given by:

(14) $$\varphi^{\prime }=Arg\;\left[r\;cos\left(\varphi \right)\;cos\left(\theta \right)+j\;r\;sin\left(\varphi \right)\right]$$

where *Arg[ ]* is the phase of the complex number between square brackets. This function is used to avoid the ambiguity of *tan^−1^( )* for angles between *π/2* and *3π/2*. In (14), the real and imaginary parts are the *r’* components over the *x’* and *y’* axes, respectively.

Finally, we must integrate (8) over all the contributions of the differential elements of the target surface (*S_T_*), and the spectral power gathered by the fiber (*P_dλ_*), is given by:

(15) $$P_{d\lambda }=\int_{S_{T}}P_{d\lambda ,dS_{T}}\left(r,\varphi ,\theta \right)dS_{T}=\int_{r=0}^{r_{T}}\int_{\varphi =0}^{2\pi }P_{d\lambda ,dS_{T}}\left(r,\varphi ,\theta \right)rd\varphi dr$$

For a circular target object, the integration limits are *0* and *r_T_*, for *r*, and *0* and *2π* for *φ*. *r_T_* is the circular target radius.

## 3. Simulations

In this section, we present simulations of the new model coded in MATLAB script. To validate the model, a study of the effects of a tilting angle on the gathered power for different target sizes and distances to the fiber end plane is presented. We also derive closed equations for the maximum allowed tilting angle to avoid changes in the power coupled to the optical fiber and for the critical distance, as in [8], versus the tilting angle. In these simulations, we consider a target emissivity of 1 and wavelength bands of 1460–1700 nm, unless otherwise stated.

### 3.1. Model Validation (Tilting Angle of 0°)

**Figure 5:**
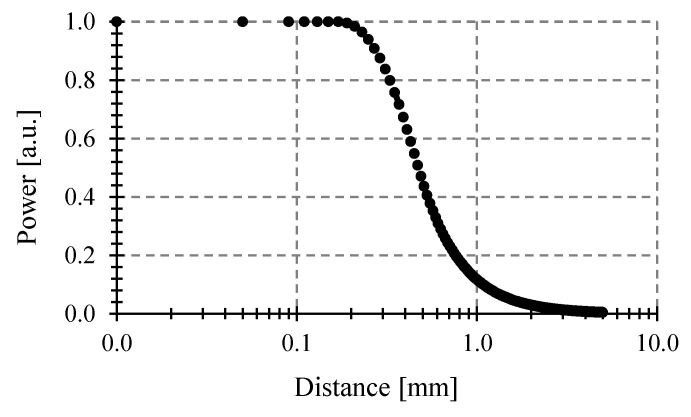
Power gathered by the pyrometer vs. distances to the target at 2000 °C, using our script with a tilting angle *θ* = 0°.

First, we consider an optical fiber with 0.29 NA and 100 μm core diameter, a target of 200 μm diameter, as in [25]. The temperature and the wavelength band are 2000 °C and 800–1700 nm, respectively. Figure 5 shows the simulations.

**Figure 6:**
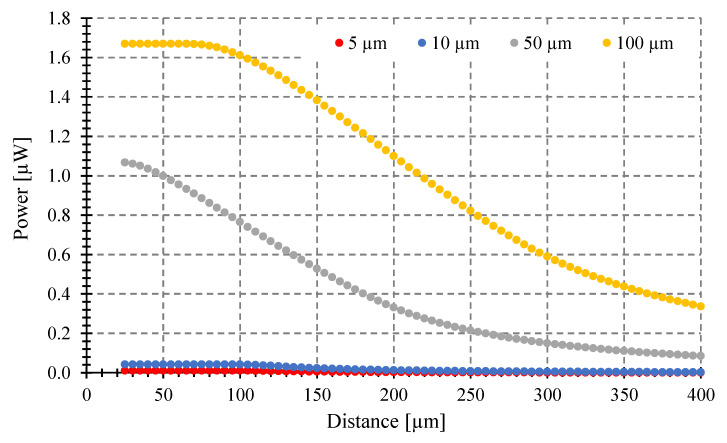
Power gathered by the pyrometer vs. distances to the target at 1000 °C, using our script with a tilting angle *θ* = 0°, for different target sizes from 5 to 100 μm diameter.

Second, we consider an optical fiber with 0.275 NA and 62.5 μm core diameter, targets of 5, 10, 50, and 100 μm diameter, as in [8]. The temperature is 1000 °C. Figure 6 shows the simulations. In both Figure 5 and Figure 6, the results agree in power levels and shape for all distances, including the critical distance where the power gathered starts to decrease, with those reported in [8].

### 3.2. Tilting Angle Effects on Power Gathered by the Pyrometer

**Figure 7:**
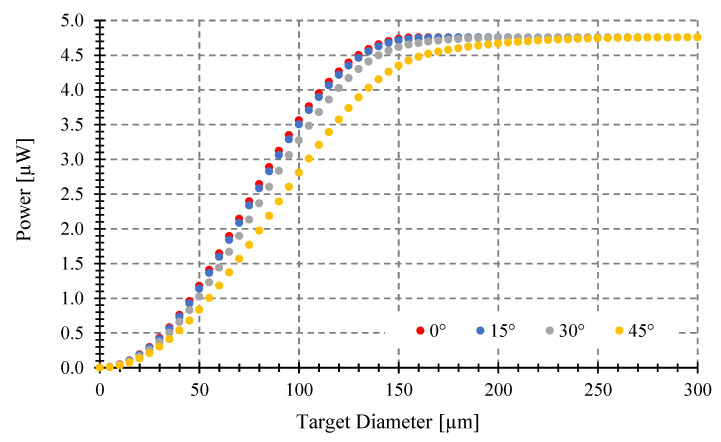
Coupled power vs. target diameter, at 1000 °C placed at 100 μm. Optical fiber with 0.29 NA and 100 μm core.

**Figure 8:**
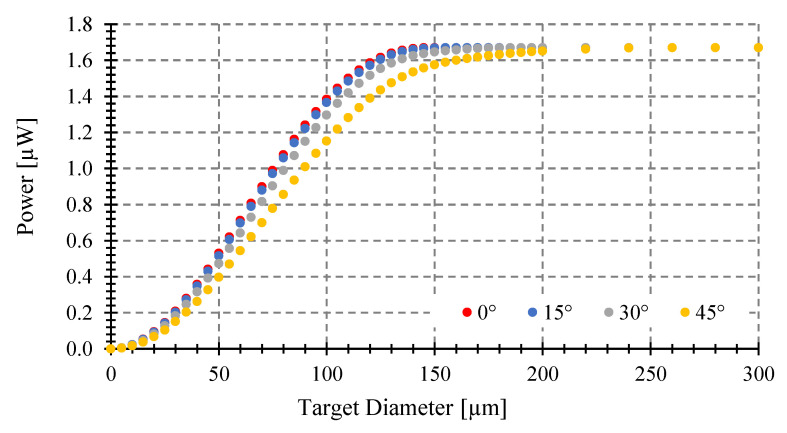
Coupled power vs. target diameter at 1000 °C placed at 150 μm. Optical fiber with 0.275 NA and 62.5 μm core. Tilting angles: 0, 15, 30, and 45°.

We first analyze the power gathered at a fixed distance from the center of the target to the fiber end of 100 μm, when changing the target diameter, for four tilting angles *θ* (0, 15, 30, 45°) (see Figure 7). We consider an optical fiber with 0.29 NA and 100 μm core diameter and targets of 5, 10, 50, and 100 μm diameter. The target temperature is 1000 °C. Then, we analyze the power gathered in the same conditions but for an optical fiber with 62.5 μm core diameter at a fixed target distance of 150 μm (see Figure 8). As we can see in Figure 7 and Figure 8, when you increase the object size, no matter which is the angle, the gathered power increases monotonically up to the same maximum value but with different slopes depending on target size. This maximum is the power gathered by the optical fiber when the object covers its entire field of view (the light cone defined by its *NA*).

As the tilting angle increases, the object diameter required to achieve the maximum gathered power increases. The greater diameter fiber gathers more power, as expected.

**Figure 9:**
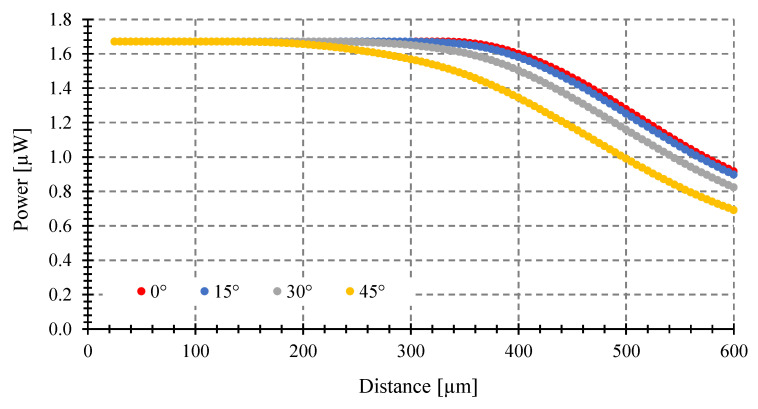
Coupled power vs. target-fiber distance at 1000 °C and a fixed target diameter of 250 μm, Optical fiber with 0.275 NA and 62.5 μm core. Tilting angles: 0, 15, 30, and 45°.

**Figure 10:**
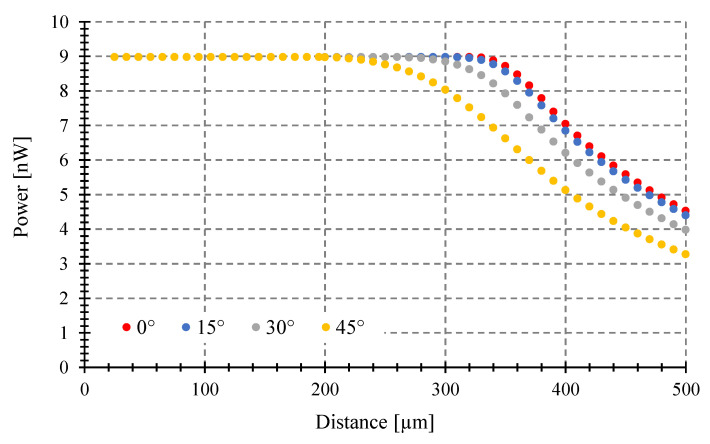
Coupled power vs. target-fiber distance at 1000 °C and a fixed target diameter of 100 μm, Optical fiber with 0.14 NA and 9 μm core. Tilting angles: 0, 15, 30, and 45°.

We examine the impact of the tilting angle on a fixed target size of 250 μm diameter when varying the distance to the target (see Figure 9), while using the same fiber and temperature as in Figure 8. Similarly, the effect is studied for a fixed target size of 100 μm diameter using an optical fiber with 0.14 NA and 9 μm core, as shown in Figure 10.

As we can see in Figure 9 and Figure 10, the power gathered by the fibers decreases monotonically for any tilting angle but with different slopes from a maximum value as the distance from the target to the fiber increases. This maximum is the coupled power of an object covering the entire field of view of the optical fiber. For a tilting angle of *θ* = 0°, the greatest distance where this happened was called the critical distance [8]. For larger tilting angles, the critical distance to have maximum power decreases. The fiber with the greater diameter and *NA* gathers more power, as expected.

Both sets of simulations show that when the field of view of the fiber is fully covered by the object, the collected power remains constant regardless of the object’s tilting angle, as expected [19]. Furthermore, the model is still valid for surfaces with reliefs other than a plane, with the condition that of each dS_T_ of the object surface, there are no rays that reflect on the surface of the object that can be coupled to the fiber.

### 3.3. Maximum Tilting Angle to Avoid Measurement Errors

The power gathered can be different depending on the tilting angle (*θ*) (see Figure 7, Figure 8, Figure 9 and Figure 10), despite having the same emissivity, wavelength band, optical fiber, etc. Therefore, a misalignment between the fiber end plane and target surface can introduce errors to the temperature measurement of a fiber-optic pyrometer. For instance, with a 100 μm target diameter and a standard multimode fiber (MMF), as shown in Figure 9, the maximum power of 1.67 μW (*θ* = 0°) is achieved at a target-fiber distance less than 200 μm when the target temperature is 1000 °C. However, if the tilting angle *θ* changes to 15, 30, and 45° at a target fiber distance of 400 μm, the collected power by the fiber decreases to 1.58, 1.5, and 1.34 μW, respectively, resulting in full-scale output errors of 5, 10, and 20%, respectively, and a maximum temperature error of around 50 °C. To prevent these errors, it is crucial to determine the maximum angle (*θ*) at which the target can be tilted while maintaining the maximum power collection at a specific distance.

**Figure 11:**
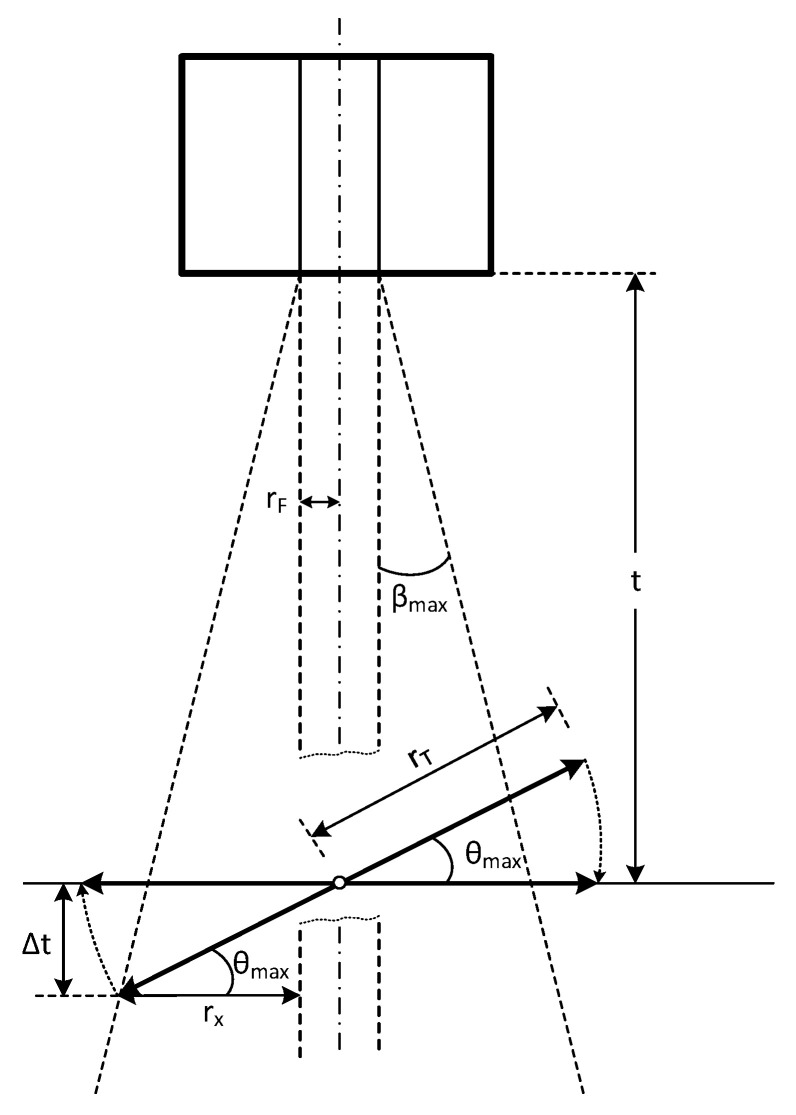
Geometry used to find the maximum tilting angle *θ_max_* to keep the maximum gathered power.

From our simulations, we show that the power collected by the fiber is independent of the angle *θ* if the diameter of the target is large enough to be fully illuminated by the maximum acceptance cone of the optical fiber, with angle *β_max_*. This phenomenon can be easily explained by utilizing the optical principle of reversibility. Nevertheless, there exists a maximum angle, denoted as *θ_max_*, beyond which, by applying the reversibility of the optical rays, the target stops being illuminated by the fiber, as depicted in Figure 11. Specifically, this limit is reached when:

(16) $$r_{x}+r_{F}=r_{T}\times cos\left(\theta_{max}\right)$$

where *r_F_* is the radius of the fiber, *r_T_* is the radius of the emitting target, and *r_x_* is given by:

(17) $$r_{x}=\left(t+r_{T}\sin\left(\theta_{max}\right)\right)\times tan\left(\beta_{max}\right)$$

where *t* is the distance from the axis of the fiber in its end plane, to the center of the target.

Substituting (17) in (16), and solving for *θ_max_*, it is found that:

(18) $$\theta_{max}={cos}^{-1}\left[\frac{r_{F}}{r_{T}}cos\left(\beta_{max}\right)+\frac{t}{r_{T}}sin\left(\beta_{max}\right)\right]-\beta_{max}$$

**Figure 12:**
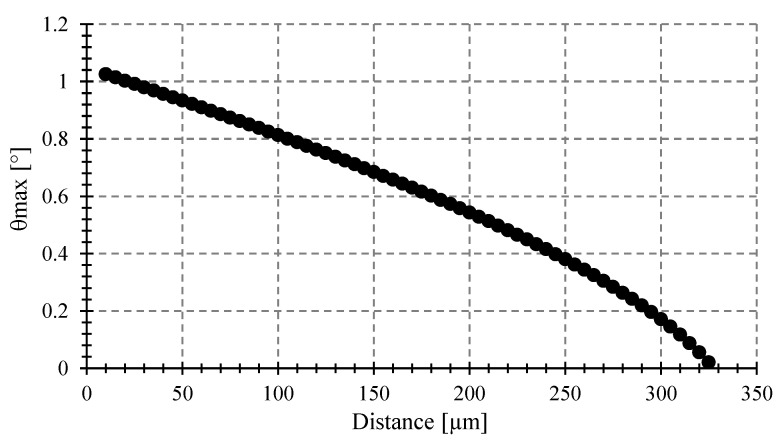
Maximum angle *θ_max_* to avoid errors vs. target-fiber distance for optical fiber with 0.275 NA and 62.5 μm core, and a circular target of 250 μm diameter.

Equation (18) gives the maximum admissible tilting angle, for a specific fiber size and *NA* and target diameter. Figure 12 shows the *θ_max_* versus distance between the target and the fiber for a target of 250 μm diameter and a multimode fiber (MMF) with 0.275 NA and 62.5 μm core. As shown in Figure 12, at larger distances, *θ_max_* decreases, eventually reaching 0 degrees at a critical distance described in [8].

### 3.4. Critical Distance as a Function of Tilting Angle

As can be seen in Figure 9 and Figure 10, the distance where the maximum power leave to be gathered (critical distance *t_c_*) is different for each tilting angle (θ). This critical distance as a function of *θ*, can be found using Equations (16) and (17), changing *θ_max_* by *θ*, and solving for *t* results λin:

(19) $$t_{c}=\frac{r_{T}\cdot cos\left(\theta +\beta_{max}\right)-r_{F}\cdot cos\left(\beta_{max}\right)}{sin\left(\beta_{max}\right)}$$

Finally, to verify the model experimental measurements will be carried out in future works using small targets as those reported in [23].

## 4. Conclusions

In this paper, a model of a fiber-optic pyrometer with a tilting angle has been proposed. The model describes pyrometers with different types of optical fibers (in terms of core diameter and numerical aperture) and for objects with different sizes placed at variable tilting angles and distances. It calculates the optical power gathered by the optical fiber. To do so, the power gathered by the optical fiber due to each differential element of the target *dS_T_*, is first calculated, and then all these differential powers are added.

The target emissive object is a surface of circular shape, with its center aligned with the axis of the optical fiber, at a known distance and size. The tilting angle is measured between the normal to the object and the fiber-optic axis. The tilting angle changes some parameters of a previously reported model [8], such as the minimum distance between the fiber end plane and each *dS_T_* (*t’*), the radius of the light projected on the fiber end plane by each *dS_T_* (*r’_βmax_*), and the distance between the center of the optical fiber and the center of circle with radius *r’_βmax_* (*r’*). This breaks the symmetry with respect to the azimuthal angle (*φ*) of each *dS_T_*, resulting in a change in the calculation algorithm and the respective integration limits. The model is described in detail, a script is implemented, and simulations are performed to verify the model.

Simulations are conducted to investigate the effect of tilting angles on the power gathered by the optical fiber when measuring the object temperature. It is observed that if the object completely covers the fiber’s field of view, the gathered power remains constant, regardless of the tilting angle, as expected [19]. This behavior can be explained by the optical principle of reversibility. Furthermore, the simulations revealed that larger tilting angles require objects of greater size and closer distances to achieve the same optical power. For instance, for a 100 µm diameter target at 400 µm distance and 1000 °C, using a standard MMF, the gathered power for tilting angles of 15, 30, and 45° is reduced with full-scale output errors of up to 20%. Such tilting can introduce measurement errors of up to 50 °C. To avoid these errors, an equation is derived to determine the maximum allowed tilting angle for a specific distance. An equation describing the critical distance, where the fiber gathered the maximum power, as a function of the tilting angle (*θ*), is also derived.

## Figures and Tables

**Table 1:** List of variables of models and their meanings.

Variable	Meaning	Variable	Meaning
*dS_T_*	Differential element of target surface	*r_T_*	Radius of the target
*r_F_*	Optical fiber (OF) core radius	*β*	Angle between the normal to *dS_T_* and the vector from *dS_T_* to each solid angle differential in the intersection of the circles with radii *r_F_* and *r_βmax_* or *r’_βmax_*
r_βmax_, r’_βmax_ *	Radius of the circle defined by the cone projection of the light, due to OF *NA*, from *dS_T_* on the fiber end plane	*dF*	Differential element of area of circle with radius *r_βmax_* or *r’_βmax_*
t, t’ *	Minimum distance from *dS_T_* to fiber end plane on each model	*u*	Radial coordinate of each differential element of area *dF*
*r*	Radial coordinate of *dS_T_* on the plane of the target	*δ*	Azimuthal coordinate of each differential element of area *dF*
*r’*	Distance between the centers of the circles with radii *r_F_* and *r_βmax_* or *r’_βmax_*	*θ*	Angle between the fiber axis and the normal to the emitting target surface
*ϕ, φ*	Azimuthal coordinate of *dS_T_* on the plane of the target on each model	*r_NA_*	Radius of the circle defined by the optical fiber field of view due numerical aperture, on the target plane
*φ’*	Angle between *r’* and *x’* axis on the fiber end plane	*β_max_*	Maximum acceptance angle of OF

* The variables *t*, *t’* and *r_βmax_*, *r’_βmax_* with and without apostrophe (new and previous model respectively), are defined in the same way but they are calculated differently.

**Table 2:** Integration limits of *u* for each *dS_T_*.

		0 < r’ < r_F_ − r’_βmax_		r_F_ − r’_βmax_ < r’ < r_F_		r_F_ < r’ < r_F_ + r’_βmax_
**r’_βmax_ < r_F_**	Int. Sit.	Circum.		Circum.	Arcs	Arcs
	u_min_	0		0	r_F_ − r’	r’ − r_F_
	u_max_	r’_βmax_		r_F_ - r’	r’_βmax_	r’_βmax_
**r_F_ < r’_βmax_ < 2r_F_**	Int. Sit.	Circum.	Arcs	Circum.	Arcs	Arcs
	u_min_	0	r_F_ − r’	0	r_F_ − r’	r’ − r_F_
	u_max_	r_F_ − r’	r_F_ + r’	r_F_ − r’	r’_βmax_	r’_βmax_
**2r_F_ < r’_βmax_**	Int. Sit.	Circum.	Arcs	Arcs		Arcs
	u_min_	0	r_F_ − r’	r’ − r_F_		r’ − r_F_
	u_max_	r_F_ − r’	r_F_ + r’	r’ + r_F_		r’_βmax_

## Data Availability

Not applicable.
